# Crystal structure of ethyl 2-(di­eth­oxy­phosphor­yl)-2-(2,3,4-tri­meth­oxy­phen­yl)acetate

**DOI:** 10.1107/S1600536814015803

**Published:** 2014-08-01

**Authors:** Moritz Schubert, Dieter Schollmeyer, Siegfried R. Waldvogel

**Affiliations:** a Johannes Gutenberg-Universität Mainz, Institut für Organische Chemie, Duesbergweg 10-14, 55128 Mainz, Germany

**Keywords:** crystal structure, Michaelis–Arbuzov reaction, phosphonoacetate, non-merohedral twin, hydrogen bonds

## Abstract

The title compound, C_17_H_27_O_8_P, was prepared by Michaelis–Arbuzov reaction of ethyl 2-bromo-2-(2,3,4-tri­meth­oxy­phen­yl)acetate and triethyl phosphite. Such compounds rarely crystallize, but single crystals were recovered after the initial oil was left for approximately 10 years. The bond angle of the *sp* ^3^-hybridized C atom connecting the benzene derivative with the phospho unit is widened marginally [112.5 (2)°]. The terminal P—O bond length of 1.464 (2) Å clearly indicates a double bond, whereas the two O atoms of the eth­oxy groups connected to the phospho­rous atom have bond lengths of 1.580 (2) Å and 1.581 (3) Å. The three meth­oxy groups emerge out of the benzene-ring plane due to steric hindrance [C—C—O—C torsion angles = −179.9 (3)°, −52.9 (4)° and 115.3 (4)°]. In the crystal, inversion dimers linked by pairs of C—H⋯O=P hydrogen bonds generate *R*_2_^2^(14) loops. The chosen crystal was modelled as a non-merohedral twin.

## Related literature   

For the complete synthesis sequence starting from the corresponding benzene derivative, see: Ianni & Waldvogel (2006 ▶). For the use of the title compound as crucial inter­mediate in a novel synthetic route for the preparation of phenanthrene carboxyl­ates, see: Schubert *et al.* (2014 ▶); Wehming *et al.* (2014 ▶). For the Michaelis–Arbuzov reaction, see: Michaelis & Kaehne (1898 ▶). For a related structure, see: Negrimovsky *et al.* (2013 ▶).
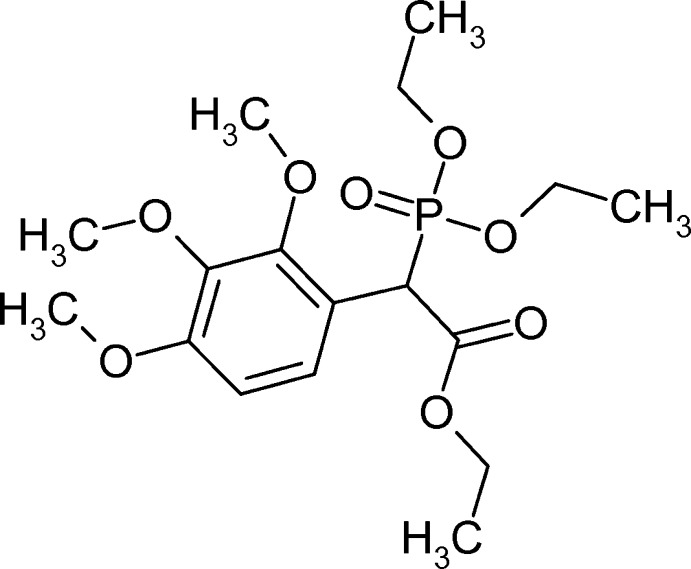


## Experimental   

### Crystal data  

C_17_H_27_O_8_P
*M* *_r_* = 390.35
Monoclinic, 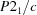

*a* = 9.6314 (14) Å
*b* = 23.749 (4) Å
*c* = 8.8155 (14) Å
β = 104.117 (4)°
*V* = 1955.5 (5) Å^3^
*Z* = 4
Mo *K*α radiation
μ = 0.18 mm^−1^
*T* = 173 K
0.64 × 0.39 × 0.06 mm

### Data collection  

Bruker SMART APEXII diffractometer
Absorption correction: multi-scan (*TWINABS*; Sheldrick, 2008*b* ▶) *T*_min_ = 0.615, *T*_max_ = 0.746
3859 measured reflections
3859 independent reflections
3033 reflections with *I* > 2σ(*I*)
*R*_int_ = 0.050

### Refinement  

*R*[*F* ^2^ > 2σ(*F* ^2^)] = 0.053
*wR*(*F* ^2^) = 0.137
*S* = 1.07
3859 reflections
236 parameters
H-atom parameters constrained
Δρ_max_ = 0.39 e Å^−3^
Δρ_min_ = −0.43 e Å^−3^

### 

Data collection: *APEX2* (Bruker, 2005 ▶); cell refinement: *SAINT* (Bruker, 2005 ▶); data reduction: *SAINT*; program(s) used to solve structure: *SHELXS2014* (Sheldrick, 2008*a* ▶); program(s) used to refine structure: *SHELXL2014* (Sheldrick, 2008*a* ▶); molecular graphics: *PLATON* (Spek, 2009 ▶); software used to prepare material for publication: *publCIF* (Westrip, 2010 ▶).

## Supplementary Material

Crystal structure: contains datablock(s) I, global. DOI: 10.1107/S1600536814015803/hb7246sup1.cif

Structure factors: contains datablock(s) I. DOI: 10.1107/S1600536814015803/hb7246Isup2.hkl

Supporting information file. DOI: 10.1107/S1600536814015803/hb7246Isup3.cml

**Fig. 1.**
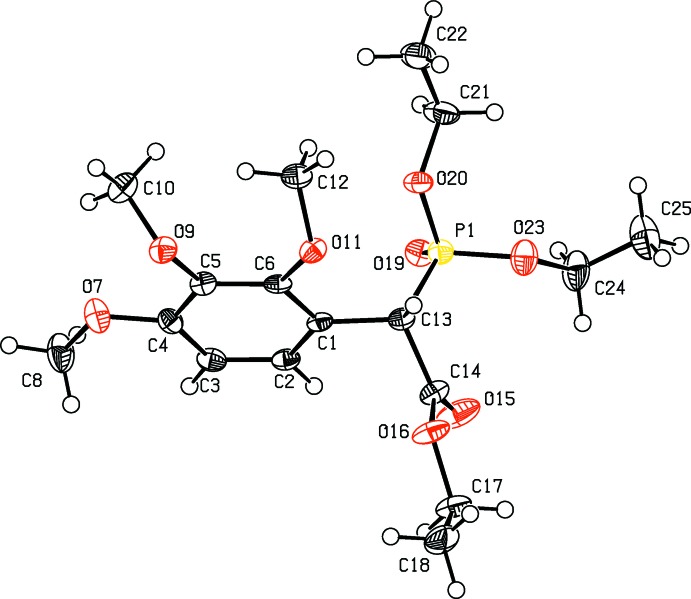
View of compound I. Displacement ellipsoids are drawn at the 50% probability level.

I . DOI: 10.1107/S1600536814015803/hb7246fig1.tif
View of compound **I**. Displacement ellipsoids are drawn at the 50% probability level.

CCDC reference: 1012505

Additional supporting information:  crystallographic information; 3D view; checkCIF report

## Figures and Tables

**Table 1:** Hydrogen-bond geometry (Å, °)

*D*—H⋯*A*	*D*—H	H⋯*A*	*D*⋯*A*	*D*—H⋯*A*
C3—H3⋯O19^i^	0.95	2.43	3.379 (4)	179

Symmetry code: (i) 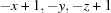.

## supplementary crystallographic information

### S1. Experimental

The title compound was prepared by heating ethyl 2-bromo-2-(2,3,4-trimethoxyphenyl)acetate (13.62 g, 40.9 mmol) with triethyl phosphite (7.4 ml, 43.4 mmol) to reflux for 2 h under inert conditions. After the reaction was cooled to room temperature H_2_O (20 ml) was added. The mixture was extracted with ethyl acetate (5 *x* 40 ml), the combined organic layer was washed with sat. NaCl solution (2 *x* 20 ml), dried over Na_2_SO_4_ and concentrated *in vacuo*. Further purification was achieved by a short-path distillation removing the excess of reagent followed by a short column chromatography using a ethyl acetate-cyclohexane mixture (40:60) as eluent. Analytically pure title compound was isolated as a colorless oil (15.67 g, 40.1 mmol, 98%). Partial crystallization of the colorless oil was observed approximately 10 years after preparation of the title compound. The storage of the material was done at ambient conditions and in absence of light. For further analytical data of the title compound, see: Ianni & Waldvogel (2006).

### S2. Refinement

Hydrogen atoms attached to carbons were placed at calculated positions with C—H = 0.95 Å (aromatic) or 0.98–0.99 Å (*sp*^3^ C-atom). All H atoms were refined in the riding-model approximation with isotropic displacement parameters (set at 1.2–1.5 times of the *U*_eq_ of the parent atom).

### Crystal data

**Table:**

C_17_H_27_O_8_P	*F*(000) = 832
*M**_r_* = 390.35	*D*_x_ = 1.326 Mg m^−^^3^
Monoclinic, *P*2_1_/*c*	Mo *K*α radiation, λ = 0.71073 Å
*a* = 9.6314 (14) Å	Cell parameters from 3917 reflections
*b* = 23.749 (4) Å	θ = 2.3–27.0°
*c* = 8.8155 (14) Å	µ = 0.18 mm^−^^1^
β = 104.117 (4)°	*T* = 173 K
*V* = 1955.5 (5) Å^3^	Plate, colourless
*Z* = 4	0.64 × 0.39 × 0.06 mm

### Data collection

**Table:**

Bruker SMART APEXII diffractometer	3859 independent reflections
Radiation source: sealed tube	3033 reflections with *I* > 2σ(*I*)
Graphite monochromator	*R*_int_ = 0.050
ω scan	θ_max_ = 26.5°, θ_min_ = 1.7°
Absorption correction: multi-scan (TWINABS; Sheldrick, 2008*b*)	*h* = −12→11
*T*_min_ = 0.615, *T*_max_ = 0.746	*k* = 0→29
3859 measured reflections	*l* = 0→11

### Refinement

**Table:**

Refinement on *F*^2^	0 restraints
Least-squares matrix: full	Hydrogen site location: inferred from neighbouring sites
*R*[*F*^2^ > 2σ(*F*^2^)] = 0.053	H-atom parameters constrained
*wR*(*F*^2^) = 0.137	*w* = 1/[σ^2^(*F*_o_^2^) + (0.0466*P*)^2^ + 2.5572*P*] where *P* = (*F*_o_^2^ + 2*F*_c_^2^)/3
*S* = 1.07	(Δ/σ)_max_ < 0.001
3859 reflections	Δρ_max_ = 0.39 e Å^−^^3^
236 parameters	Δρ_min_ = −0.43 e Å^−^^3^

### Special details

**Table:**

Geometry. All e.s.d.'s (except the e.s.d. in the dihedral angle between two l.s. planes) are estimated using the full covariance matrix. The cell e.s.d.'s are taken into account individually in the estimation of e.s.d.'s in distances, angles and torsion angles; correlations between e.s.d.'s in cell parameters are only used when they are defined by crystal symmetry. An approximate (isotropic) treatment of cell e.s.d.'s is used for estimating e.s.d.'s involving l.s. planes.
Refinement. Refined as a 2-component twin.

### Fractional atomic coordinates and isotropic or equivalent isotropic displacement parameters (Å^2^)

**Table:**

	*x*	*y*	*z*	*U*_iso_*/*U*_eq_	
P1	0.82647 (10)	0.11292 (4)	0.68241 (11)	0.0215 (2)	
C1	0.5353 (3)	0.11793 (14)	0.6724 (4)	0.0187 (7)	
C2	0.4706 (4)	0.06673 (14)	0.6276 (4)	0.0211 (7)	
H2	0.5183	0.0332	0.6703	0.025*	
C3	0.3376 (4)	0.06270 (15)	0.5217 (4)	0.0231 (8)	
H3	0.2952	0.0269	0.4929	0.028*	
C4	0.2680 (4)	0.11141 (14)	0.4587 (4)	0.0212 (7)	
C5	0.3294 (4)	0.16442 (14)	0.5046 (4)	0.0196 (8)	
C6	0.4629 (3)	0.16751 (13)	0.6109 (4)	0.0174 (7)	
O7	0.1382 (2)	0.11366 (10)	0.3518 (3)	0.0306 (7)	
C8	0.0703 (4)	0.06102 (17)	0.3011 (6)	0.0395 (11)	
H8A	−0.0211	0.0679	0.2253	0.059*	
H8B	0.1325	0.0385	0.2519	0.059*	
H8C	0.0530	0.0406	0.3914	0.059*	
O9	0.2567 (3)	0.21360 (10)	0.4542 (3)	0.0243 (6)	
C10	0.2349 (4)	0.22605 (17)	0.2889 (5)	0.0342 (9)	
H10A	0.1826	0.2616	0.2649	0.051*	
H10B	0.3280	0.2293	0.2630	0.051*	
H10C	0.1795	0.1957	0.2272	0.051*	
O11	0.5264 (3)	0.21800 (9)	0.6647 (3)	0.0223 (5)	
C12	0.5414 (4)	0.25940 (15)	0.5502 (5)	0.0304 (9)	
H12A	0.5879	0.2931	0.6036	0.046*	
H12B	0.5999	0.2439	0.4836	0.046*	
H12C	0.4465	0.2694	0.4857	0.046*	
C13	0.6843 (3)	0.12212 (14)	0.7830 (4)	0.0195 (8)	
H13	0.6941	0.1609	0.8288	0.023*	
C14	0.7003 (4)	0.08058 (15)	0.9180 (4)	0.0249 (8)	
O15	0.7525 (3)	0.03478 (12)	0.9248 (3)	0.0454 (8)	
O16	0.6416 (3)	0.10238 (11)	1.0281 (3)	0.0321 (6)	
C17	0.6348 (5)	0.06604 (17)	1.1595 (5)	0.0350 (9)	
H17A	0.7324	0.0556	1.2190	0.042*	
H17B	0.5813	0.0311	1.1215	0.042*	
C18	0.5600 (5)	0.0985 (2)	1.2603 (5)	0.0431 (11)	
H18A	0.5532	0.0755	1.3504	0.065*	
H18B	0.6141	0.1329	1.2969	0.065*	
H18C	0.4637	0.1085	1.1999	0.065*	
O19	0.8192 (3)	0.06344 (10)	0.5818 (3)	0.0257 (6)	
O20	0.8143 (3)	0.17162 (10)	0.5957 (3)	0.0244 (6)	
C21	0.9160 (4)	0.18363 (17)	0.5007 (5)	0.0352 (10)	
H21A	0.9029	0.1564	0.4134	0.042*	
H21B	1.0153	0.1801	0.5654	0.042*	
C22	0.8901 (4)	0.24207 (19)	0.4380 (6)	0.0437 (11)	
H22A	0.9575	0.2507	0.3741	0.066*	
H22B	0.7918	0.2451	0.3737	0.066*	
H22C	0.9039	0.2688	0.5252	0.066*	
O23	0.9696 (3)	0.11834 (11)	0.8151 (3)	0.0331 (7)	
C24	1.0793 (4)	0.07506 (18)	0.8476 (6)	0.0399 (11)	
H24A	1.0805	0.0544	0.7504	0.048*	
H24B	1.0588	0.0478	0.9243	0.048*	
C25	1.2197 (4)	0.1021 (2)	0.9113 (6)	0.0513 (13)	
H25A	1.2946	0.0732	0.9337	0.077*	
H25B	1.2396	0.1288	0.8345	0.077*	
H25C	1.2180	0.1222	1.0080	0.077*	

### Atomic displacement parameters (Å^2^)

**Table:**

	*U*^11^	*U*^22^	*U*^33^	*U*^12^	*U*^13^	*U*^23^
P1	0.0230 (4)	0.0194 (4)	0.0225 (5)	0.0009 (4)	0.0062 (4)	0.0003 (4)
C1	0.0236 (17)	0.0192 (17)	0.0154 (16)	0.0019 (14)	0.0091 (14)	0.0032 (15)
C2	0.0276 (19)	0.0157 (17)	0.0223 (18)	0.0023 (13)	0.0105 (16)	0.0033 (15)
C3	0.0277 (19)	0.0161 (17)	0.028 (2)	−0.0004 (14)	0.0125 (16)	−0.0002 (15)
C4	0.0197 (17)	0.0223 (18)	0.0231 (17)	−0.0006 (14)	0.0080 (14)	−0.0035 (16)
C5	0.0235 (18)	0.0165 (17)	0.0205 (18)	0.0036 (13)	0.0088 (14)	−0.0008 (14)
C6	0.0263 (18)	0.0126 (16)	0.0168 (17)	−0.0005 (13)	0.0120 (14)	−0.0009 (14)
O7	0.0223 (13)	0.0267 (14)	0.0401 (16)	0.0004 (11)	0.0020 (11)	−0.0056 (12)
C8	0.031 (2)	0.032 (2)	0.049 (3)	−0.0051 (17)	−0.005 (2)	−0.007 (2)
O9	0.0270 (13)	0.0172 (12)	0.0287 (13)	0.0058 (10)	0.0071 (11)	0.0028 (11)
C10	0.037 (2)	0.035 (2)	0.028 (2)	0.0052 (17)	0.0032 (19)	0.0073 (19)
O11	0.0300 (13)	0.0140 (11)	0.0215 (13)	−0.0008 (10)	0.0036 (11)	0.0003 (11)
C12	0.035 (2)	0.0218 (19)	0.035 (2)	−0.0032 (16)	0.0086 (18)	0.0081 (17)
C13	0.0247 (18)	0.0182 (17)	0.0174 (19)	0.0009 (14)	0.0085 (15)	−0.0007 (14)
C14	0.035 (2)	0.0213 (19)	0.0179 (19)	0.0010 (15)	0.0054 (16)	0.0030 (15)
O15	0.071 (2)	0.0349 (17)	0.0366 (17)	0.0255 (15)	0.0260 (17)	0.0162 (14)
O16	0.0522 (17)	0.0288 (14)	0.0207 (14)	0.0094 (12)	0.0191 (13)	0.0070 (12)
C17	0.047 (2)	0.037 (2)	0.025 (2)	0.0016 (18)	0.0158 (19)	0.0115 (19)
C18	0.046 (3)	0.065 (3)	0.020 (2)	0.003 (2)	0.014 (2)	0.006 (2)
O19	0.0284 (14)	0.0243 (13)	0.0263 (13)	−0.0002 (11)	0.0101 (12)	−0.0030 (11)
O20	0.0274 (14)	0.0223 (13)	0.0267 (13)	0.0014 (10)	0.0127 (11)	0.0064 (11)
C21	0.036 (2)	0.034 (2)	0.043 (2)	0.0025 (17)	0.024 (2)	0.011 (2)
C22	0.036 (2)	0.046 (3)	0.054 (3)	0.004 (2)	0.020 (2)	0.022 (2)
O23	0.0285 (14)	0.0308 (15)	0.0366 (16)	0.0065 (11)	0.0015 (12)	−0.0036 (13)
C24	0.030 (2)	0.032 (2)	0.051 (3)	0.0120 (17)	−0.003 (2)	0.001 (2)
C25	0.031 (2)	0.045 (3)	0.071 (3)	0.0013 (19)	−0.001 (2)	−0.006 (3)

### Geometric parameters (Å, º)

**Table:**

P1—O19	1.464 (2)	C12—H12C	0.9800
P1—O20	1.580 (2)	C13—C14	1.524 (5)
P1—O23	1.581 (3)	C13—H13	1.0000
P1—C13	1.817 (3)	C14—O15	1.194 (4)
C1—C2	1.379 (5)	C14—O16	1.341 (4)
C1—C6	1.408 (4)	O16—C17	1.458 (4)
C1—C13	1.529 (5)	C17—C18	1.488 (6)
C2—C3	1.392 (5)	C17—H17A	0.9900
C2—H2	0.9500	C17—H17B	0.9900
C3—C4	1.384 (5)	C18—H18A	0.9800
C3—H3	0.9500	C18—H18B	0.9800
C4—O7	1.370 (4)	C18—H18C	0.9800
C4—C5	1.408 (5)	O20—C21	1.463 (4)
C5—O9	1.378 (4)	C21—C22	1.492 (6)
C5—C6	1.396 (5)	C21—H21A	0.9900
C6—O11	1.377 (4)	C21—H21B	0.9900
O7—C8	1.431 (4)	C22—H22A	0.9800
C8—H8A	0.9800	C22—H22B	0.9800
C8—H8B	0.9800	C22—H22C	0.9800
C8—H8C	0.9800	O23—C24	1.452 (4)
O9—C10	1.450 (5)	C24—C25	1.478 (6)
C10—H10A	0.9800	C24—H24A	0.9900
C10—H10B	0.9800	C24—H24B	0.9900
C10—H10C	0.9800	C25—H25A	0.9800
O11—C12	1.441 (4)	C25—H25B	0.9800
C12—H12A	0.9800	C25—H25C	0.9800
C12—H12B	0.9800		
			
O19—P1—O20	115.34 (14)	C1—C13—P1	112.5 (2)
O19—P1—O23	114.73 (15)	C14—C13—H13	107.4
O20—P1—O23	103.56 (15)	C1—C13—H13	107.4
O19—P1—C13	117.50 (15)	P1—C13—H13	107.4
O20—P1—C13	98.87 (14)	O15—C14—O16	124.2 (3)
O23—P1—C13	104.72 (16)	O15—C14—C13	126.2 (3)
C2—C1—C6	118.7 (3)	O16—C14—C13	109.6 (3)
C2—C1—C13	121.9 (3)	C14—O16—C17	117.0 (3)
C6—C1—C13	119.4 (3)	O16—C17—C18	106.8 (3)
C1—C2—C3	122.0 (3)	O16—C17—H17A	110.4
C1—C2—H2	119.0	C18—C17—H17A	110.4
C3—C2—H2	119.0	O16—C17—H17B	110.4
C4—C3—C2	119.2 (3)	C18—C17—H17B	110.4
C4—C3—H3	120.4	H17A—C17—H17B	108.6
C2—C3—H3	120.4	C17—C18—H18A	109.5
O7—C4—C3	125.5 (3)	C17—C18—H18B	109.5
O7—C4—C5	114.3 (3)	H18A—C18—H18B	109.5
C3—C4—C5	120.2 (3)	C17—C18—H18C	109.5
O9—C5—C6	119.0 (3)	H18A—C18—H18C	109.5
O9—C5—C4	121.3 (3)	H18B—C18—H18C	109.5
C6—C5—C4	119.6 (3)	C21—O20—P1	117.9 (2)
O11—C6—C5	122.4 (3)	O20—C21—C22	108.6 (3)
O11—C6—C1	117.3 (3)	O20—C21—H21A	110.0
C5—C6—C1	120.2 (3)	C22—C21—H21A	110.0
C4—O7—C8	116.8 (3)	O20—C21—H21B	110.0
O7—C8—H8A	109.5	C22—C21—H21B	110.0
O7—C8—H8B	109.5	H21A—C21—H21B	108.4
H8A—C8—H8B	109.5	C21—C22—H22A	109.5
O7—C8—H8C	109.5	C21—C22—H22B	109.5
H8A—C8—H8C	109.5	H22A—C22—H22B	109.5
H8B—C8—H8C	109.5	C21—C22—H22C	109.5
C5—O9—C10	115.7 (3)	H22A—C22—H22C	109.5
O9—C10—H10A	109.5	H22B—C22—H22C	109.5
O9—C10—H10B	109.5	C24—O23—P1	123.4 (3)
H10A—C10—H10B	109.5	O23—C24—C25	108.8 (3)
O9—C10—H10C	109.5	O23—C24—H24A	109.9
H10A—C10—H10C	109.5	C25—C24—H24A	109.9
H10B—C10—H10C	109.5	O23—C24—H24B	109.9
C6—O11—C12	117.7 (3)	C25—C24—H24B	109.9
O11—C12—H12A	109.5	H24A—C24—H24B	108.3
O11—C12—H12B	109.5	C24—C25—H25A	109.5
H12A—C12—H12B	109.5	C24—C25—H25B	109.5
O11—C12—H12C	109.5	H25A—C25—H25B	109.5
H12A—C12—H12C	109.5	C24—C25—H25C	109.5
H12B—C12—H12C	109.5	H25A—C25—H25C	109.5
C14—C13—C1	110.9 (3)	H25B—C25—H25C	109.5
C14—C13—P1	111.0 (2)		
			
C6—C1—C2—C3	1.1 (5)	C6—C1—C13—C14	138.9 (3)
C13—C1—C2—C3	−177.2 (3)	C2—C1—C13—P1	82.2 (4)
C1—C2—C3—C4	0.2 (5)	C6—C1—C13—P1	−96.1 (3)
C2—C3—C4—O7	178.9 (3)	O19—P1—C13—C14	73.8 (3)
C2—C3—C4—C5	−1.6 (5)	O20—P1—C13—C14	−161.5 (2)
O7—C4—C5—O9	5.2 (5)	O23—P1—C13—C14	−54.8 (3)
C3—C4—C5—O9	−174.3 (3)	O19—P1—C13—C1	−51.1 (3)
O7—C4—C5—C6	−178.8 (3)	O20—P1—C13—C1	73.6 (3)
C3—C4—C5—C6	1.6 (5)	O23—P1—C13—C1	−179.7 (2)
O9—C5—C6—O11	−1.6 (5)	C1—C13—C14—O15	96.2 (4)
C4—C5—C6—O11	−177.6 (3)	P1—C13—C14—O15	−29.7 (5)
O9—C5—C6—C1	175.7 (3)	C1—C13—C14—O16	−81.6 (4)
C4—C5—C6—C1	−0.3 (5)	P1—C13—C14—O16	152.6 (3)
C2—C1—C6—O11	176.4 (3)	O15—C14—O16—C17	−3.6 (6)
C13—C1—C6—O11	−5.3 (4)	C13—C14—O16—C17	174.2 (3)
C2—C1—C6—C5	−1.1 (5)	C14—O16—C17—C18	−177.4 (3)
C13—C1—C6—C5	177.3 (3)	O19—P1—O20—C21	−55.7 (3)
C3—C4—O7—C8	−0.4 (5)	O23—P1—O20—C21	70.5 (3)
C5—C4—O7—C8	−179.9 (3)	C13—P1—O20—C21	178.1 (3)
C6—C5—O9—C10	115.3 (4)	P1—O20—C21—C22	−176.8 (3)
C4—C5—O9—C10	−68.7 (4)	O19—P1—O23—C24	−6.6 (4)
C5—C6—O11—C12	−52.9 (4)	O20—P1—O23—C24	−133.2 (3)
C1—C6—O11—C12	129.7 (3)	C13—P1—O23—C24	123.7 (3)
C2—C1—C13—C14	−42.8 (4)	P1—O23—C24—C25	150.9 (3)

### Hydrogen-bond geometry (Å, º)

**Table:**

*D*—H···*A*	*D*—H	H···*A*	*D*···*A*	*D*—H···*A*
C3—H3···O19^i^	0.95	2.43	3.379 (4)	179

Symmetry code: (i) −*x*+1, −*y*, −*z*+1.
