# Supplementary figures and images for: Crystal structure of ethyl 2-(di­eth­oxy­phosphor­yl)-2-(2,3,4-tri­meth­oxy­phen­yl)acetate
Source: Acta Crystallogr Sect E Struct Rep Online. 2014 Aug 1;70(Pt 9):o897–8. doi: 10.1107/S1600536814015803 (PMC4186086; doi:10.1107/S1600536814015803)

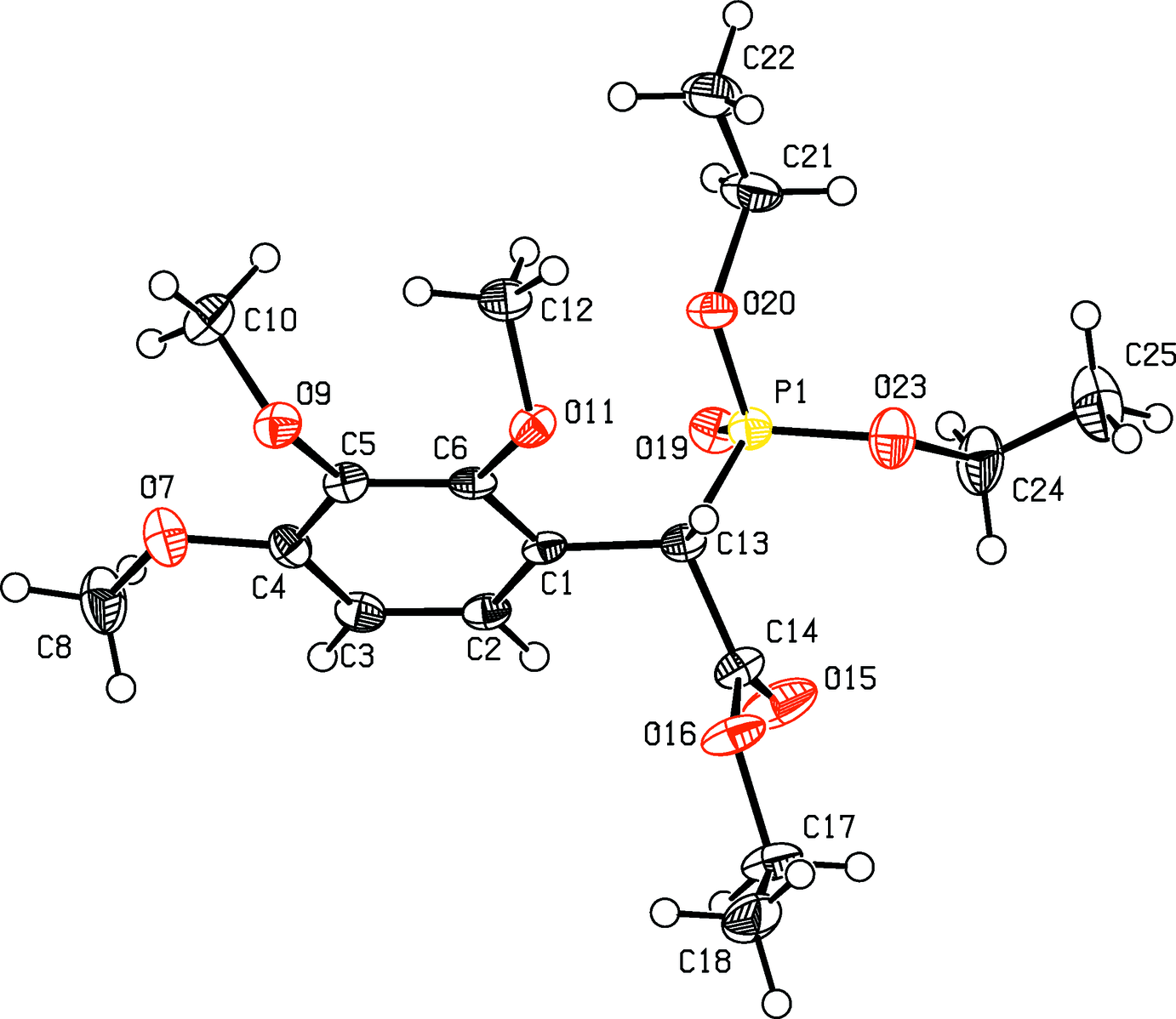

Supplement: Supplementary file 4 [file e-70-0o897-fig1.tif]
